# Modifications to a vegan artificial honey result in inhibitory activity against *Mycobacterium abscessus*

**DOI:** 10.1371/journal.pone.0356687

**Published:** 2026-09-10

**Authors:** Victoria C. Nolan, James Harrison, Jonathan A. G. Cox

**Affiliations:** School of Life and Health Sciences, Aston University, Aston Triangle, Birmingham, United Kingdom; St Petersburg Pasteur Institute, RUSSIAN FEDERATION

## Abstract

The multidrug resistant *Mycobacterium abscessus* is an opportunistic pathogen of increasing concern, causing infections of skin and soft tissue as well as pulmonary, it can be life-threatening for immunocompromised individuals. Current treatment strategies are ineffective and new approaches to combat infections are imperative. The antimicrobial potential of Manuka honey has been recently demonstrated to be effective against *M. abscessus,* but the potential to improve its efficacy as well as reduce demand on a natural resource have not yet been explored. The development of medical honey paves the way for generation of a synthetic honey, but this information is largely hidden behind patents and also not explored from a synergist perspective of combined activity of MGO, H_2_O_2_ and defensin-1. Through addition of key enzymes or components and antimicrobial susceptibility testing, we have demonstrated that a vegan honea, containing no bee derived component results in antimicrobial activity against *M. abscessus*, causing not only growth inhibition but bactericidal activity. Both MGO and H_2_O_2_ are demonstrated to be relevant to the killing effect against *M. abscessus*.

## 1 Introduction

The increasing threat of antimicrobial resistance is continuing to pose a large burden on health care in the modern world. One organism of increasing concern is the intrinsically multi-drug resistant *Mycobacterium abscessus*. Ubiquitous in the environment, known for causing infections of the skin and soft tissue as well as pulmonary infection, it is of significant importance to immunocompromised individuals [1,2]. The current approaches to treating infections are inadequate and often unsuccessful, resulting in a desperate need for new treatment strategies or novel approaches for combating these infections [3–5].

The exploration into natural products for antimicrobial therapy is growing, with a significant interest in manuka honey. The understanding of what makes a manuka honey and non-manuka honey antimicrobial has been well studied in recent years, with methylglyoxal (MGO), hydrogen peroxide (H_2_O_2_) and bee derived defensin-1 identified as the main components [6,7]. The combination of these, along with other properties of honey such as high sugar content and osmotic pressure result in honey having a broad spectrum of activity [8].

Understanding how these key antimicrobials are generated and utilizing this information has a great potential for furthering honey as a treatment strategy. The generation of MGO is through a non-enzymatic conversion of dihydroxyacetone, a component of *Leptospermum* sp. nectar, in a process referred to as honey ripening [9,10]. As MGO concentrations increase, the DHA decreases, resulting in an irreversible conversion achieved through a dehydration reaction [11]. The resulting MGO concentration, Leptosperin content, DHA and hydroxymethylfurfural content are used to grade the manuka honey with an MGO rating, determining the mg/kg concentrations of these components present. The occurrence of H_2_O_2_ in honey is due to the addition of invertase, diastase and glucose oxidase, which are produced by the honeybee during nectar harvest [6]. This results in the breakdown of larger disaccharides into monosaccharides and with the addition of oxygen, glucose oxidase catalyses the oxidation of glucose to D-glucono-δ-lactone and H_2_O_2_ [6,12]. The presence of the defensin-1 protein is due to direct addition from the honeybee as an immune response. Typically present at low levels, between 0.04 µg/mL and 5.17 µg/mL in Slovakian honeys, its presence is influenced by honeybee health [7,13].

This has led to the development of medical based honeys, usually focused on one of the components and not in combination. Importantly, the knowledge of how to generate these components in a non-honey background has been rather unexplored. With the decline in honeybee populations and the potential to make a more potent or targeted medical honey utilising this knowledge, we explored the possibility of generating an antimicrobial honey using a vegan honea background, containing no honeybee derived components. Through this, we have demonstrated the potential for developing an antimicrobial non-honeybee vegan honea that has antimicrobial activity against *M. abscessus*.

## 2 Materials and methods

### 2.1 Chemicals and reagents

All chemicals and reagents were obtained from Sigma-Aldrich or Melford Laboratories, unless otherwise stated. As the core for our component-based design we used the vegan non-honey bee derived honey alternative ‘Vegan Honea’ (Plant Based Artisans, UK). The product mimics the natural consistency, carbohydrate structure (sugar and inulin) and taste of honey while containing no bee-derived components, ingredients list: Sugar (60 g per 100 g), Inulin, Apple Juice, Natural Flowers, Lemon Juice, Natural flavours, Molasses. The Honea was stored in the dark at room temperature. Prior to testing, 1 g/mL stocks of vegan Honea in sterile distilled water were made and filtered through a 0.22 µm filter.

### 2.2 Growth of *Mycobacteria abscessus* cultures

*M. abscessus* NCTC 13031 was used for all susceptibility testing. *M. abscessus* cultures were grown from glycerol stocks (stored at −80 °C), in Middlebrook 7H9 broth, supplemented with glycerol (1% w/v) and Tween80 (0.05% w/v), at 37 °C, for 72 h in an orbital shaker at 180 rpm.

### 2.3 Broth microdilutions and determination of MIC and MBC

All broth microdilutions were prepared by adding all samples to be tested into a 96 well plate with 7H9 broth and inoculated with 5 µL *M. abscessus* OD_600nm_=0.1. These were incubated at 37 °C for 96 h, with OD reads taken every 24 h. After 96 h, 5 µL of all wells were transferred onto Middlebrook 7H11 agar supplemented with glycerol (1% w/v) and incubated at 37 °C for a further 72 h. The minimum inhibitory concentration (MIC) was determined as the minimum concentration required to inhibit the growth of *M. abscessus* and the minimum bactericidal concentration (MBC) was determined as the minimum concentration where no growth of *M. abscessus* was observed on solid media.

### 2.4 Broth microdilution of H_2_O_2_, MGO, defensin-1 and vegan Honea

To assess the impact of each component’s antimicrobial activity against *M. abscessus*, broth microdilutions were conducted as described in section 2.3. Concentrations of H_2_O_2_ were prepared from a 2 M stock of H_2_O_2_ in sterile distilled water and final concentrations of 20 mM, 10 mM, 5 mM and 0 mM (control) H_2_O_2_ were used for testing. For MGO, a 500 mM stock was prepared in sterile distilled water and final concentrations of 5 mM, 2.5 mM, 1.25 mM, 0.625 mM and 0 mM (control) MGO were used for testing. Bee defensin-1 (Kingfisher Biotech) was prepared in sterile phosphate buffered saline to a stock concentration of 1 mg/mL, final concentrations for testing were 5 µg/mL and 10 µg/mL. Vegan Honea stocks were prepared to 1 g/mL (w/v) in distilled water and sterilised using a 0.22 µM filter. Final concentrations for testing were 0.476 g/mL, 0.238 g/mL and 0.119 g/mL.

### 2.5 Modified vegan Honea to generate H_2_O_2_

To determine antimicrobial activity broth microdilutions were prepared as described in section 2.3. 1 U/mL invertase and glucose oxidase was prepared with 1 g/mL vegan Honea in distilled water and sterilised using a 0.22 µm filter. The vegan Honea containing invertase and glucose oxidase was serially diluted in 7H9 broth to final concentrations of 0.476 g/mL, 0.238 g/mL, 0.119 g/mL, 0.0595 g/mL, 0.029 g/mL, 0.014 g/mL, 0.007 g/mL and 0 g/mL of vegan Honea.

### 2.6 Modified vegan Honea to generate MGO

To generate the MGO within the vegan Honea a 10 mg/mL stock of DHA was prepared in distilled water and used to make a 1 g/mL stock of vegan Honea containing 8 mg/mL DHA. A control of DHA alone was prepared to a stock concentration of 8 mg/mL. All samples were sterilised using a 0.22 µm filter. These were prepared in duplicate and stored at either 37 °C or 4 °C prior to testing, with the initial incubation time of 2 h prior to broth microdilution set up for day 0 samples. All samples were then stored at either 37 °C or 4 °C for a total of 112 days with broth microdilutions every 14 days to monitor changes in antimicrobial activity. Broth microdilutions were conducted for vegan Honea with DHA or DHA alone as described in section 2.3. Due to the dilution of DHA stock solutions for testing, the estimated concentration of DHA was used to demonstrate the highest concentration tested and the subsequent more dilute concentrations. The final concentrations tested in each broth microdilution were 0.476 g/mL vegan Honea with 1.9 mg/mL DHA, 1.9 mg/mL DHA only, 0.238 g/mL vegan Honea with 0.9 mg/mL DHA, 0.9 mg/mL DHA only, 0.119 g/mL vegan Honea with 0.49 mg/mL DHA and 0.49 mg/mL DHA only.

### 2.7 Data analysis

All data was processed using Excel and subsequently analysed using GraphPad Prism 8. Statistical analysis included data normality checks, followed by one-way ANOVA with Dunnett’s multiple comparison.

## 3 Results

### 3.1 Exploration of H_2_O_2_, MGO, defensin-1 and sugar content on antimicrobial impact against *M. abscessus* growth

**Fig 1:**
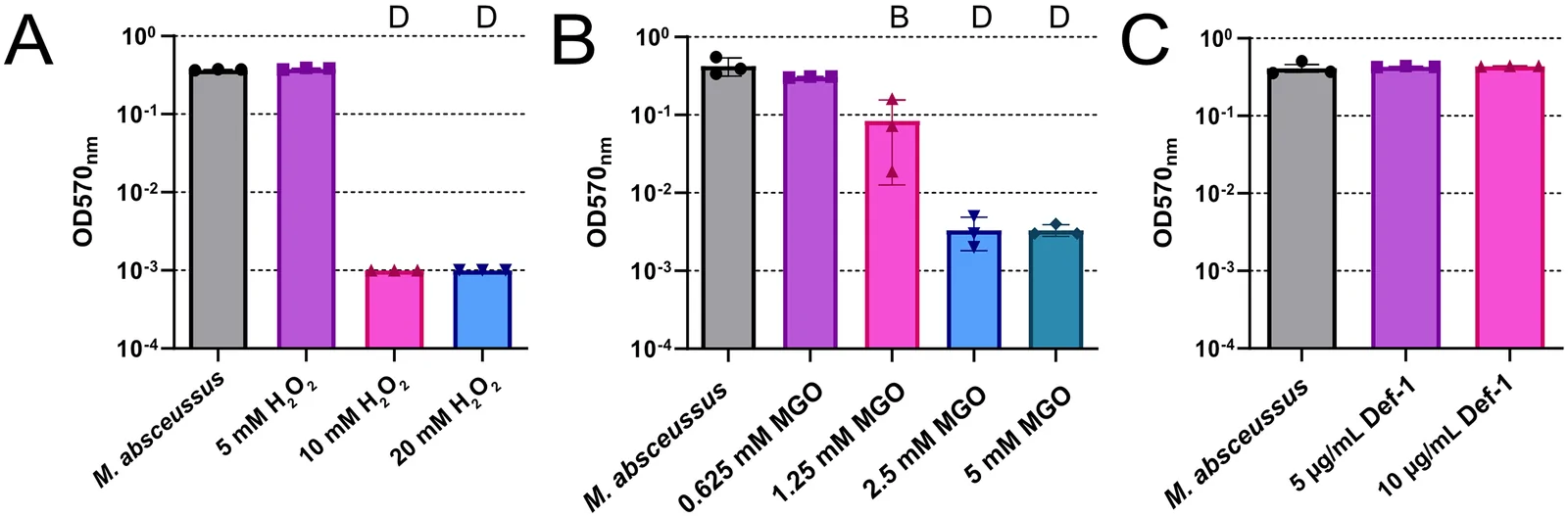
Growth inhibition of *M. abscessus* NCTC 13031 exposed to individual antimicrobial components of honey (n = 3). A) H_2_O_2_ shows inhibitory activity against *M. abscessus* at 10 mM or higher (One-way ANOVA, P=<0.0001.). B) MGO prevents growth of *M. abscessus* at 2.5 mM or higher, with negative impact on growth at 1.25 mM but not complete growth inhibition (One-way ANOVA, P=<0.0001). C) Honeybee derived defensin-1 shows no inhibitory activity against *M. abscessus* (One-way ANOVA, P = 0.9850). D) Vegan Honea has some impact on the growth of *M. abscessus* but growth was observed for all concentrations tested (One-way ANOVA, P = 0.0011) [14].

In order to establish which components of honey exert antimicrobial activity against *M. abscessus*, each one was tested using the broth microdilution assays (Fig 1). The concentrations of H_2_O_2_ required to cause growth inhibition of *M. abscessus* were 10 mM or higher (Fig 1A). The 5 mM H_2_O_2_ had no impact on the growth of *M. abscessus*, with no significant difference observed between 5 mM H_2_O_2_ versus no treatment (Dunnet’s multiple comparison, P = 0.0582). The bactericidal activity also reflected this, with no growth observed for 20 mM or 10 mM H_2_O_2_ when plated onto solid media and visible growth was observed for *M. abscessus* exposed to 5 mM H_2_O_2_.

MGO showed dose-dependent inhibitory activity against *M. abscessus*, with 0.625 mM MGO showing some reduction in growth, however this was not significant (Dunnett’s multiple comparison, P = 0.1035) (Fig 1B). All other concentrations of MGO showed significant impact on the growth of *M. abscessus*. Growth was observed for 1.25 mM MGO but this was only observed for the last 24 h of the experiment, suggesting this concentration had a negative impact on *M. abscessus* and all higher concentrations showed complete growth inhibition. The bactericidal activity also reflected the inhibitory activity with no visible growth observed for *M. abscessus* exposed to 5 mM MGO and 2.5 mM MGO, with growth observed for *M. abscessus* exposed to both 1.25 mM MGO and 0.625 mM MGO.

Defensin-1 showed no inhibitory activity against *M. abscessus* at either concentration tested (One-way ANOVA, P = 0.9850) (Fig 1C). No bactericidal activity was observed.

Vegan Honea impacted the growth of *M. abscessus* but no MIC was established (Fig 1D). The highest concentration of 0.476 g/mL showed significant difference in response compared to no treatment (Dunnett’s multiple comparison, P = 0.0006), as well as 0.238 g/mL (Dunnett’s multiple comparison, P = 0.0358). There was no difference for 0.119 g/mL and no treatment (Dunnett’s multiple comparison, P = 0.5519). No bactericidal activity was observed for vegan Honea against *M. abscessus*.

### 3.2 H_2_O_2_ generation in vegan Honea produces antimicrobial activity

**Fig 2:**
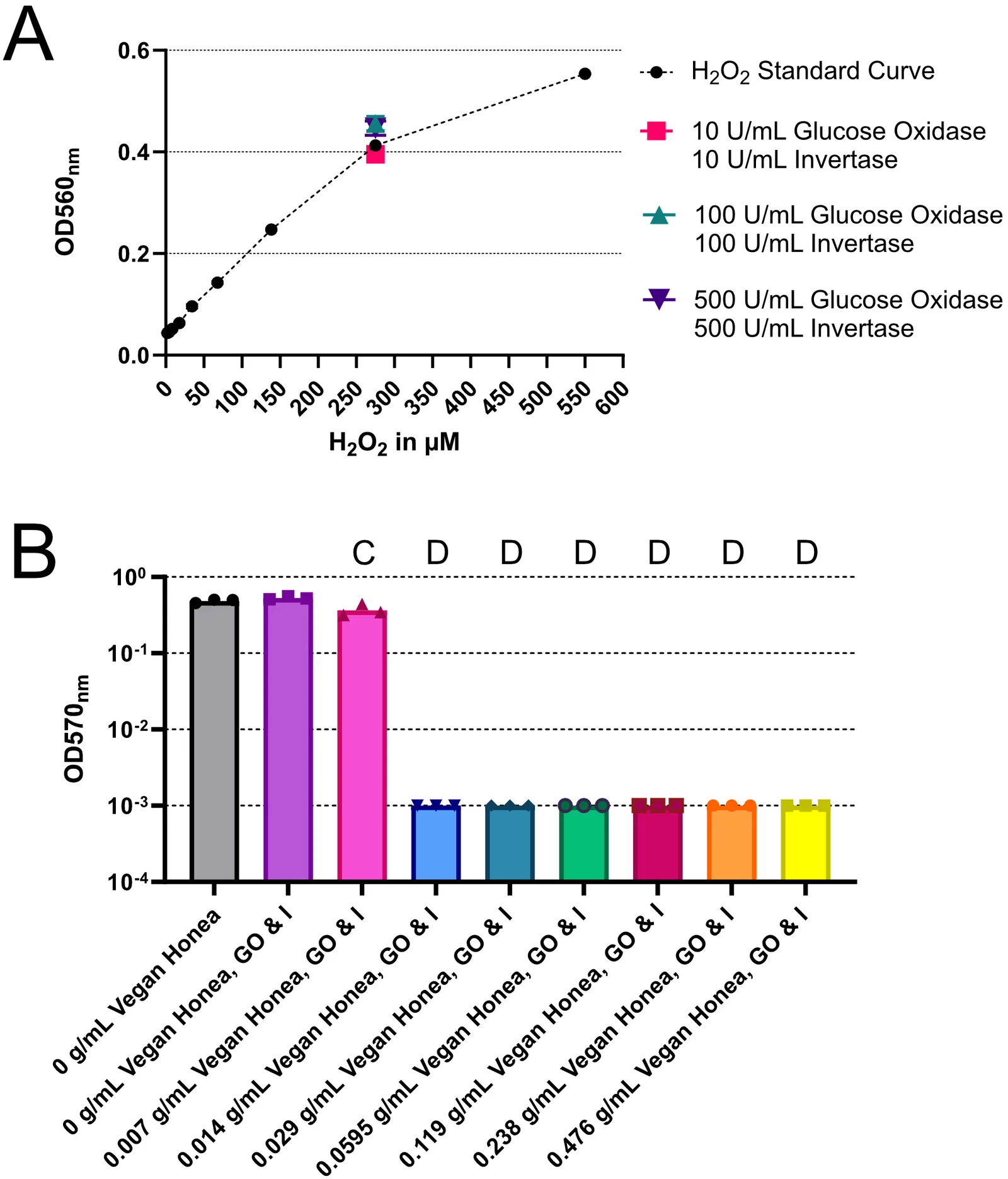
H_2_O_2_ generation in vegan Honea using glucose oxidase and invertase results in antimicrobial activity against *M. abscessus* (n = 3). Vegan Honea with glucose oxidase and invertase results in improved antimicrobial activity against *M. abscessus*. Concentrations of 0.014 g/mL vegan Honea with glucose oxidase and invertase or higher show growth inhibition (One-way ANOVA, P=<0.0001).

H_2_O_2_ generation in the vegan Honea was achieved by the addition of glucose oxidase and invertase enzymes. 1 Unit/mL (U/mL) of both enzymes were added to the stock of 1 g/mL vegan Honea for broth microdilution experiments. This addition of glucose oxidase and invertase resulted in growth inhibition by 0.014 g/mL vegan Honea, where previously no complete growth inhibition was observed for vegan Honea alone (Fig 1D and 2). This was also reflected in bactericidal activity, where vegan Honea alone had no bactericidal activity, but vegan Honea with glucose oxidase and invertase demonstrated no *M. abscessus* growth with 0.014 g/mL vegan Honea.

### 3.3 Vegan Honea modified to generate MGO shows increased antimicrobial activity against *M. abscessus*

**Fig 3:**
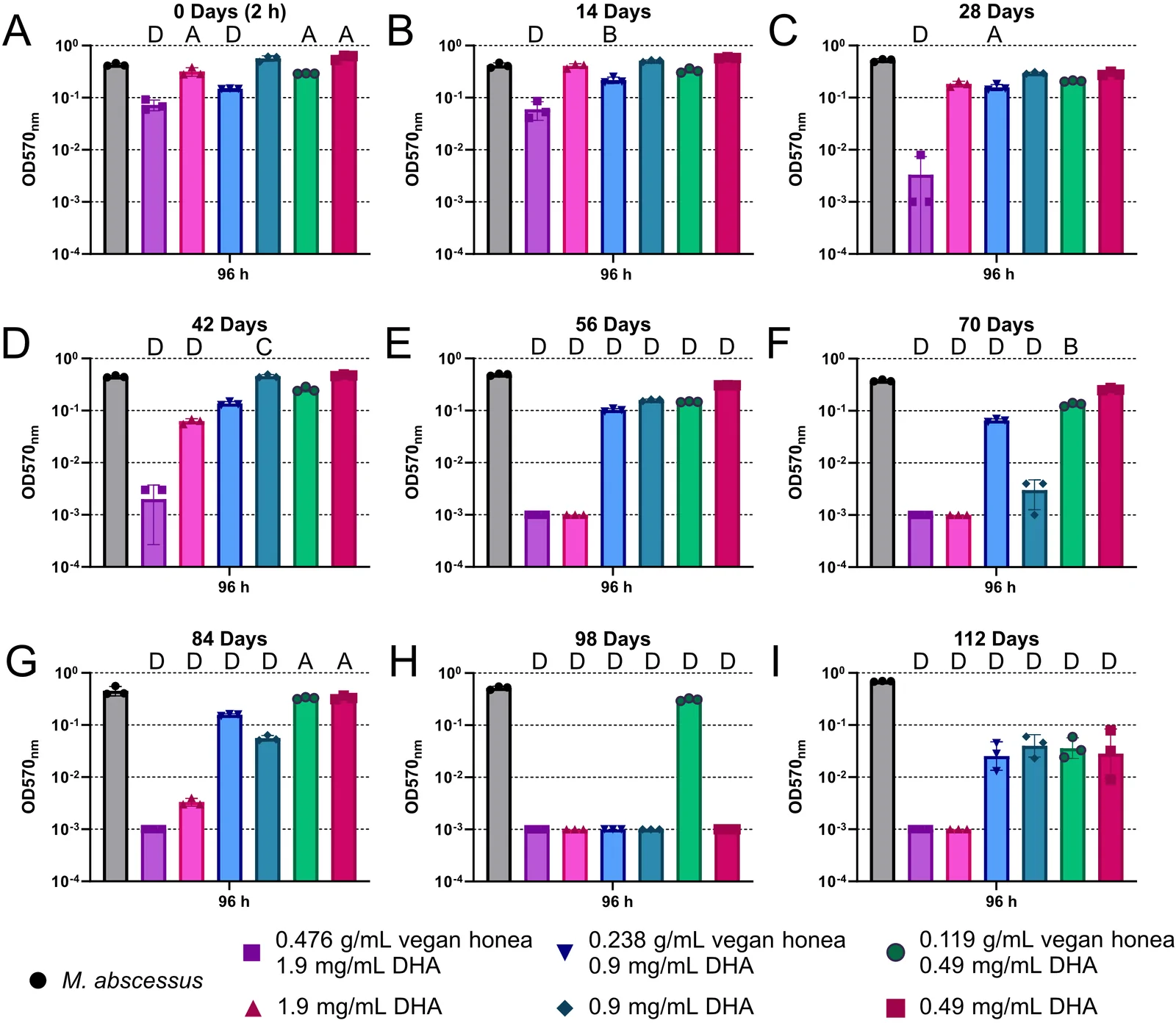
Vegan Honea with DHA stored at 37 °C for 112 days shows improved activity against *M. abscessus* NCTC 13031 (n = 3). All concentrations tested were dilutions of the same stock of 1 g/mL vegan honea with 8 mg/mL DHA or a stock solution of 8 mg/mL only. The concentrations reported are estimated concentrations of the diluted stock. An increase in antimicrobial activity of vegan Honea containing DHA can be observed over time. End point data, taken at 96 h, was used for statistical analysis to determine the impact of vegan Honea with DHA on the growth of *M. abscessus*. A) No complete inhibitory activity of vegan Honea with DHA at any concentration tested against *M. abscessus* after 2 h incubation. B) Vegan Honea with DHA stored for 14 days showing no complete inhibition of *M. abscessus* at any concentration. C) Complete growth inhibition of *M. abscessus* by 0.476 g/mL vegan Honea with 1.9 mg/mL DHA stored for 28 days prior to testing. Dose-dependent inhibition was observed for all other concentrations tested. D) Vegan Honea with DHA after 42 days of storage exhibiting complete growth inhibition by 0.476 g/mL vegan Honea with 1.9 mg/mL DHA. Dose-dependent inhibition was observed for all other concentrations tested. E) Vegan Honea with DHA stored for 56 days prior to testing showed complete growth inhibition of *M. abscessus* with 0.476 g/mL vegan Honea with 1.9 mg/mL DHA and 1.9 mg/mL DHA only. F) After 70 days of storage, 0.476 g/mL vegan Honea with 1.9 mg/mL DHA, 1.9 mg/mL DHA and 0.9 mg/mL DHA alone showed complete growth inhibition to *M. abscessus*. G) The storage of vegan Honea with DHA for 84 days prior to testing showed *M. abscessus* growth inhibition for 0.476 g/mL vegan Honea with 1.9 mg/mL DHA and 1.9 mg/mL only. H) All concentrations tested completely inhibited the growth of *M. abscessus* apart from 0.119 g/mL vegan Honea with 0.49 g/mL DHA after 98 days of storage. I) After 112 days of storage, all concentrations of vegan Honea with DHA and DHA alone inhibited *M. abscessus* apart from 0.119 g/mL vegan Honea with 0.49 mg/mL DHA and 0.49 mg/mL DHA only.

**Fig 4:**
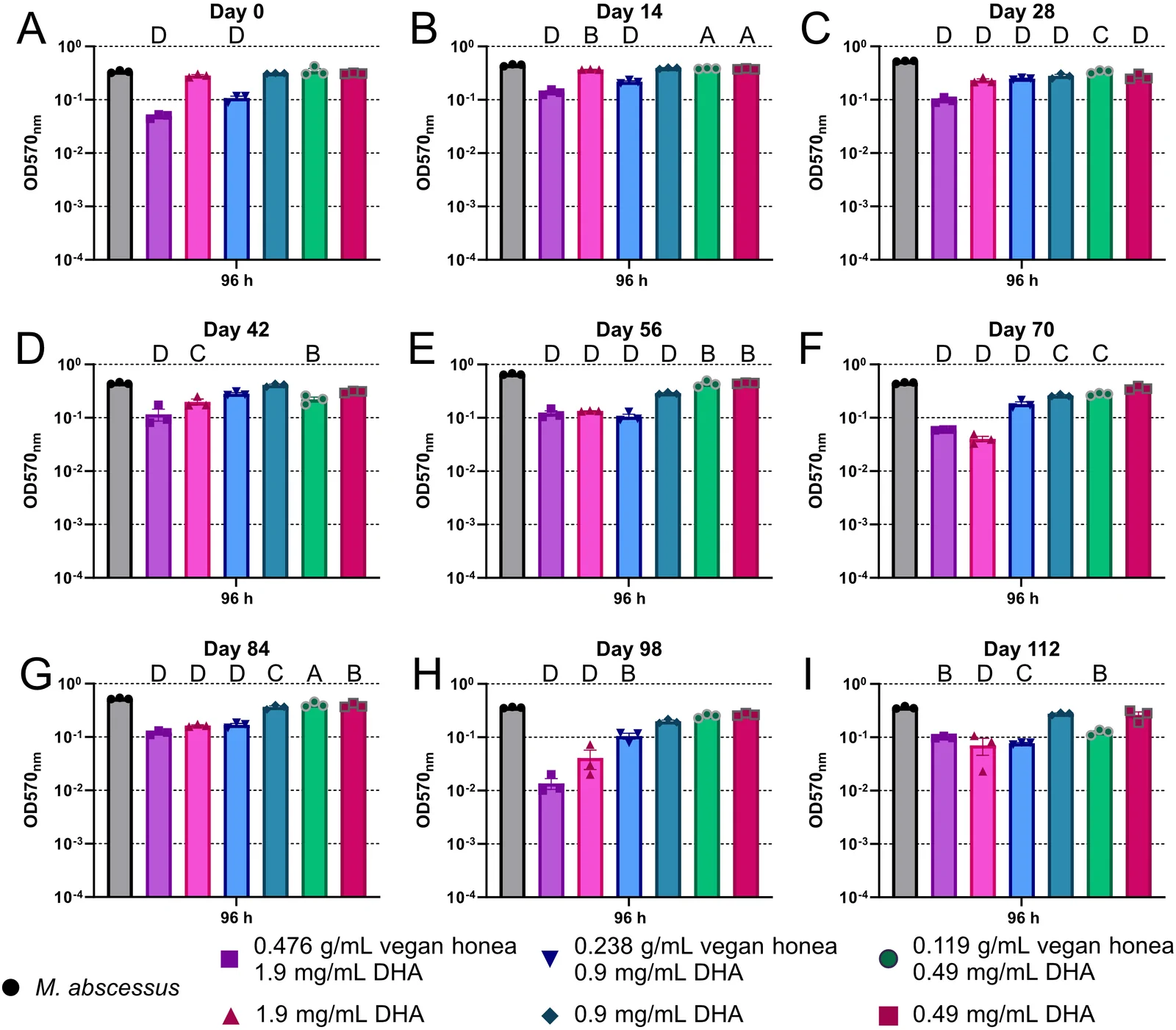
Vegan Honea with DHA stored at 4 °C for 112 days shows improved activity against *M. abscessus* NCTC 13031 (n = 3). All concentrations tested were dilutions of the same stock of 1 g/mL vegan honea with 8 mg/mL DHA or a stock solution of 8 mg/mL only. The concentrations reported are estimated concentrations of the diluted stock. No increased antimicrobial activity of vegan Honea containing DHA was observed, regardless of storage time. End point data, taken at 96 h, was used for statistical analysis to determine the impact of vegan Honea with DHA on the growth of *M. abscessus*. A) Vegan Honea with DHA stored for 2 h showed a reduction in growth for *M. abscessus* but no complete growth inhibition. B) Vegan Honea with DHA stored for 14 days prior to testing maintaining the reduction in growth for higher concentrations of vegan Honea and DHA, but no complete growth inhibition was observed. C) Storage of vegan Honea with DHA for 28 days showing no complete growth inhibition against *M. abscessus*. D) No complete growth inhibition was observed for vegan Honea with DHA against *M. abscessus* after 42 days of storage. E) Storage of vegan Honea for 56 days maintaining no compelte growth inhibtiion but a dose-dependent reduction in growth of *M. abscessus* was observed. F) A reduction in growth of *M. abscessus* for 0.476 g/mL vegan Honea with 1.9 mg/mL DHA and 1.9 mg/mL DHA alone stored for 70 days but no complete growth inhibition. G) No complete growth inhibition was observed for *M. abscessus* exposed to vegan Honea with DHA stored for 84 days. H) Vegan Honea with DHA stored for 98 days showed complete growth inhibition against *M. abscessus* at 0.476 g/mL vegan Honea with 1.9 mg/mL DHA. All other concentrations showed a dose-depenedent response on the growth of *M. abscessus*. I) Loss of complete growth inhibition of *M. abscessus* by vegan Honea with DHA after 112 days of storage.

MGO generation within the vegan Honea was achieved through addition of DHA and stored for up to 112 days to facilitate conversion. All concentrations of DHA reported were dilutions of the same 8 mg/mL DHA stock and the estimated concentrations present after subsequent dilution at time of testing. Variations in both inhibitory and bactericidal activity were observed over time, with improved antimicrobial activity observed for vegan Honea containing DHA stored at 37 °C compared to samples stored at 4 °C (Figs 3 and 4).

Initially, no complete growth inhibition was observed for vegan Honea with DHA stored at 37 °C for any concentrations tested on days 0 and 14, but growth of *M. abscessus* was negatively impacted in a dose-depenedent manner (Fig 3 A and B). DHA alone at the lower dilutions of 0.49 mg/mL and 0.9 mg/mL DHA showed improved growth at day 0 and day 14 compared to *M. abscessus* only (Fig 3 A and B), demonstrating DHA alone was not inhibitory to *M. abscessus*. No bactericidal activity was observed for any concentration tested at day 0 and 14. After 28 days of storage at 37 °C complete growth inhibition was observed for 0.476 g/mL vegan Honea with 1.9 mg/mL DHA (Fig 3 C). Interestingly, the same concentration of 1.9 mg/mL DHA alone did not impact the growth of *M. abscessus*. All other concentrations of vegan Honea with DHA and DHA only showed a reduction in growth compared to *M. abscessus* only, but these were not considered inhibitory. No bactericidal activity was observed for any concentration.

After 42 days of storage at 37 °C the growth inhibition was maintained with only 0.476 g/mL vegan Honea with 1.9 mg/mL DHA showing no growth (Fig 3 D). However, *M. abscessus* exposed to 1.9 mg/mL DHA exhibited a reduction in growth compared to the growth of *M. abscessus* only. After 56 days of storage at 37 °C, 0.476 g/mL vegan Honea with 1.9 mg/mL DHA remained inhibitory (Fig 3 E). The control of 1.9 mg/mL DHA alone also showed inhibitory activity (Fig 3 E). All other concentrations showed a reduction in growth compared to *M. abscessus* only, but these were not inhibitory. No bactericidal activity was identified for any concentration after storage at either 42 days or 56 days.

After 70 days of storage at 37 °C, 3 concentrations showed complete growth inhibition to *M. abscessus* (Fig 3 F). These were 0.476 g/mL vegan Honea with 1.9 mg/mL DHA, 1.9 mg/mL DHA alone and 0.9 mg/mL DHA alone. All other concentrations maintained a reduction in growth compared to *M. abscessus*. Interestingly, there was no bactericidal activity for 0.476 g/mL vegan Honea with 1.9 mg/mL DHA but bactericidal activity was observed for 1.9 mg/mL DHA only.

The antimicrobial activity observed after 84 days of storage at 37 °C maintained inhibition against *M. abscessus* at 0.476 g/mL vegan Honea with 1.9 mg/mL DHA as well as the control of 1.9 mg/mL DHA alone (Fig 3 G). However, a loss of inhibition was observed for 0.9 mg/mL DHA alone. All other concentrations were not inhibitory to *M. abscessus* but a reduction in growth was observed. A change in bactericidal activity was also observed, with 0.476 g/mL vegan Honea with 1.9 mg/mL DHA and 1.9 mg/mL DHA alone being bactericidal.

A significant difference in activity was observed after 98 days of storage at 37 °C, with almost all concentrations being inhibitory towards *M. abscessus* (Fig 3 H). The only concentration that did not inhibit *M. abscessus* was 0.119 g/mL vegan Honea with 0.49 mg/mL DHA. The bactericidal activity remained the same with only 0.476 g/mL vegan Honea with 1.9 mg/mL DHA and 1.9 mg/mL DHA alone being bactericidal.

After 112 days of storage at 37 °C, almost all concentrations remained inhibitory towards *M. abscessus* (Fig 3 I). The concentration of 0.119 g/mL vegan Honea with 0.49 mg/mL DHA remained not inhibitory and a loss of inhibition was observed for 0.49 mg/mL DHA alone. The bactericidal activity was also maintained, with 0.476 g/mL vegan Honea with 1.9 mg/mL DHA and 1.9 mg/mL DHA only being bactericidal.

The antimicrobial activity of vegan Honea with DHA stored at 4 °C against *M. abscessus* differed to that stored at 37 °C and did not have the same inhibitory effect (Fig 4). There was no complete growth inhibition observed for any concentration tested until day 98 of storage, which was 0.476 g/mL vegan Honea with 1.9 mg/mL DHA and after that growth was observed again. The vegan Honea with DHA had a greater inhibitory activity against *M. abscessus* than compared to DHA alone for all days tested. No bactericidal activity was observed for any day of testing at any concentration.

### 3.4 Combination of vegan Honea with DHA, glucose oxidase and invertase maintains antimicrobial activity against *M. abscessus*

**Fig 5:**
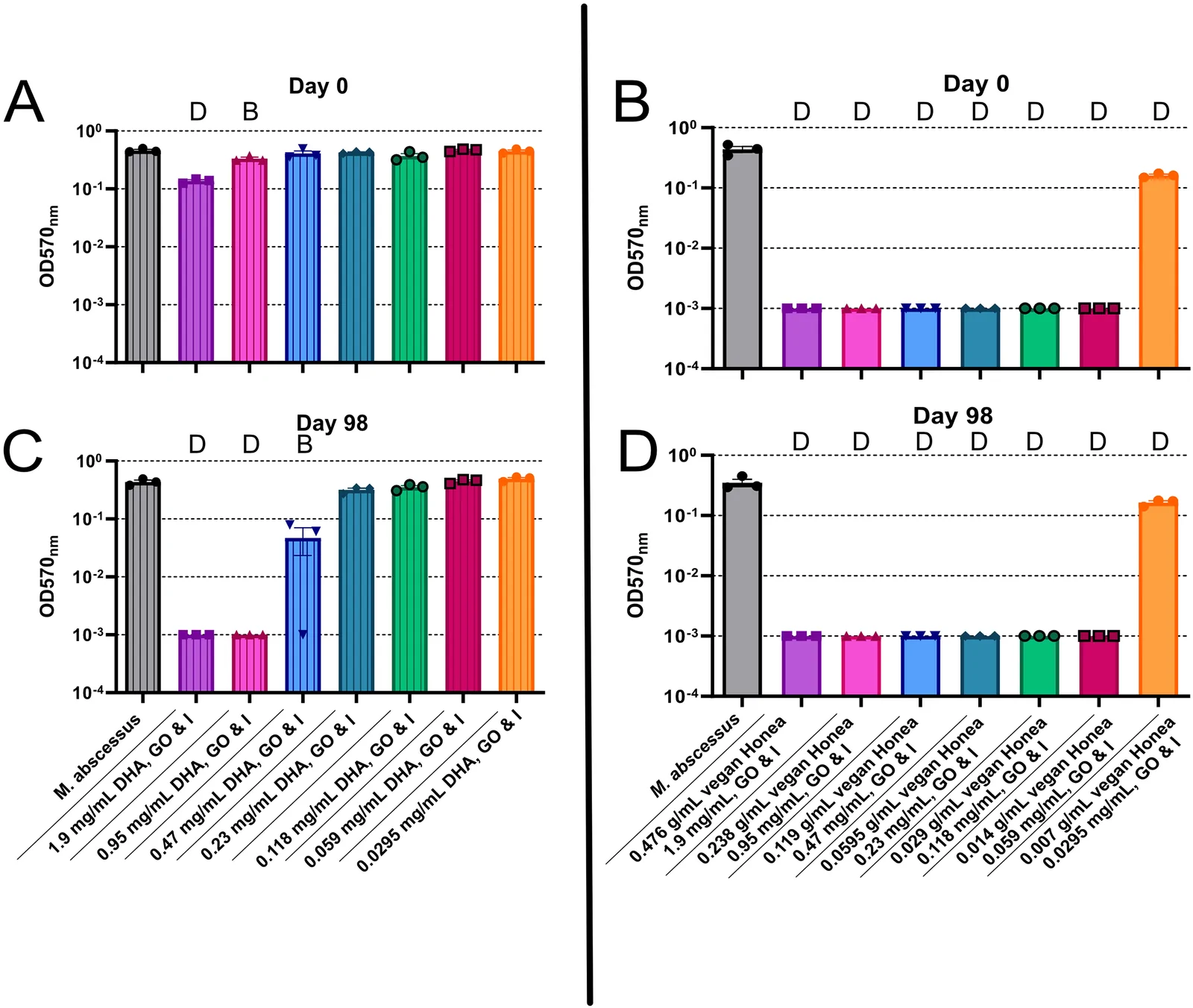
Adding specific precursors of Manuka honey in combination to a vegan honey alternative (Honea) gives significant enhancement of antimicrobial activity against *M. abscessus.* Each component was made to a stock concentration and the reported concentrations were as a result of broth microdilution. A) DHA in sterile distilled water incubated for 2 h at 37 °C with the addition of glucose oxidase and invertase shows no growth inhibition of *M. abscessus*. A reduction in growth of 1.9 mg/mL DHA with glucose oxidase and invertase shows growth reduction compared to all other concentrations. B) DHA in sterile distilled water incubated for 98 days at 37 °C with the addition of glucose oxidase and invertase shows growth inhibition of *M. abscessus* at 0.95 mg/mL DHA or higher. Reduction in growth was observed for 0.47 mg/mL DHA. C) Vegan Honea with DHA incubated for 2 h at 37 °C, with the addition of glucose oxidase and invertase, results in growth inhibition of *M. abscessus*. D) Vegan Honea incubated for 98 days with DHA at 37 °C maintains the same growth inhibition of *M. abscessus* compared to 0 days of Honea incubation.

To observe if improved antimicrobial activity could be achieved against *M. abscessus* with the combination of vegan Honea with the addition of DHA, glucose oxidase and invertase, broth microdilutions were conducted. The stock concentrations of DHA, glucose oxidase and invertase were the same as reported in previous sections, with the calculated concentrations reported to demonstrate the dilutions achieved after broth microdilution. Due to the time required for DHA conversion to MGO, two time points of Honea maturation were selected, day 0 and day 98 with Honea incubation at 37 °C. The addition of glucose oxidase and invertase resulted in complete growth inhibition with 0.014 g/mL vegan Honea for both day 0 and day 98 (Fig 5A and B). This was the same as observed for vegan Honea with glucose oxidase and invertase without the DHA (Fig 2B). Controls of DHA in sterile distilled water incubated at 37 °C for 0 and 98 days, with glucose oxidase and invertase, show no improved antimicrobial activity compared to DHA alone (Fig 5 B and C).

## 4 Discussion

Honey engineered for use within the medical industry has become of great interest in recent years [15]. Several medicinal honey’s have been produced with differing mechanisms of action, that are often protected under patents. However, the 3 main basis for producing a medical grade honey are MGO, H_2_O_2_ and defensin-1 [16–19]. Often, the antimicrobial component chosen for the basis of producing a medical honey influences its activity. Those based on production of hydroxyl radicals, such as surgihoney, have the most improved efficacy, followed by manuka based medical honey’s such as Medihoney and lastly Revamil based on defensin-1 [20]. Since these components cannot be controlled for in commercially produced honey, coupled with the decline of honeybees in recent years threatening the future of honey production, we propose that these components could be combined in a vegan Honea to produce an effective antimicrobial honey alternative, to combat difficult to treat infections.

Pulmonary and soft tissue infections caused by *M. abscessus* are of increasing concern, with ineffective treatments and limited new therapies, there is now an urgent requirement for exploration into novel strategies. Considering the recent findings that manuka honey can inhibit the growth of *M. abscessus* [14], we wanted to explore the components that give rise to honeys antimicrbobial activity, MGO,H_2_O_2_ and defensin-1 and their role in inhibiting *M. abscessus*. Understanding which components are important for the inhibition of *M. abscessus* would help to develop the antimicrobial vegan Honea.

The concentrations of H_2_O_2_ identified within natural honey samples range between 0.01 mM and 2.68 mM [6,21]. These concentrations are varied and the lower concentrations are not inhibitory on their own. Previous observations identified that 1.25 mM and 2.5 mM H_2_O_2_ are required to inhibit the growth of *Escherichia coli* and *Bacillus subtilis*, respectively [6]. However, the concentration of H_2_O_2_ required for inhibition of *M. abscessus* was 10 mM (Fig 1), much higher than what is naturally found in honey. This is a relatively high concentration and it is likely required due to the presence of *katG* in *M. abscessus*, which is a catalase-peroxidase enzyme, resulting in the detoxification of H_2_O_2_ [22,23].

To explore H_2_O_2_ generation within a vegan Honea and its impact on antimicrobial activity, both invertase and glucose oxidase enzymes were added to the vegan Honea. The addition of both enzymes resulted in a significant increase in antimicrobial activity. Vegan Honea alone has no complete growth inhibtion against *M. abscessus*, however the addition of glucose oxidase and invertase resulted in no growth with 0.014 g/mL vegan honea containing both enzymes (Fig 1D and 2). This was also reflected in the bactericidal activity, with no growth observed for 0.014 g/mL vegan Honea with glucose oxidase and invertase, whereas vegan Honea alone showed *M. abscessus* growth. Considering 10 mM H_2_O_2_ was required to cause inhibitory activity to *M. abscessus*, it is likely high concentrations of H_2_O_2_ were generated in the vegan Honea. Quantification of H_2_O_2_ generation was not possible for this study so H_2_O_2_ concentrations could not be varfied.

Inhibitory profiles similar to the H_2_O_2_ were likewise observed for MGO. The concentration of MGO required for complete growth inhibition of *M. abscessus* was 2.5 mM (Fig 1B). This is higher than previous observations of the Gram-positive *B. subtilis* and *Staphylococcus aureus*, which required 0.8 mM and 1.2 mM MGO, respectively, to inhibit growth and the Gram-negative *E. coli* and *Pseudomonas aeruginosa* which required 1.0 mM and 1.2 mM, respectively to cause growth inhibition [24]. Bactericidal activity was also observed for 2.5 mM MGO for *M. abscessus*. Concentrations of 2 mM MGO have been demonstrated to cause damage to external structures and cell membranes in both *B. subtilis* and *E. coli* [24]. If MGO causes damage to the outer mycobacterial membrane, accumulation of MGO could occur within the periplasmic space. This accumulation would result in damage to the cytoplasmic membrane and could result in cell death. Furthermore, it has been identified that MGO is not recognised by drug efflux pumps in other bacterial isolates [25]. This suggests that, providing mycobacteria do not have specific MGO efflux pumps, after entry into the cell it is very unlikely MGO would be exported out, improving its antimicrobial action and preventing one of the main resistance mechanisms employed by mycobacteria. Additionally, the activity previously observed by manuka honey against *M. abscessus* used relatively low grades of manuka honey ranging from MGO40 to MGO83, suggesting that higher grades of manuka honey that can be rated up to MGO2200+, would likely be more effective against *M. abscessus* [14].

To further explore the potential generation of MGO within a vegan Honea, resulting in antimicrobial activity, DHA was added to the vegan Honea. This was selected as a more natural process that would mimic MGO generation within the Honea, rather than just addition of MGO. The conversion of DHA to MGO occurs over time and is influenced by temperature, with higher temperature resulting in higher turnover [26]. Therefore, 2 different temperatures were selected for the storage of the vegan Honea with DHA, these were 37 °C and 4 °C. Over the duration of the 112 days of honea maturation stored at 37 °C, antimicrobial activity against *M.abscessus* increased, this was also in a dose-dependent manner. Higher concentrations of vegan honea with DHA and DHA alone showed improved inhibitory and bactericidal activity with all concentrations showing inhibition by day 112, suggesting sufficient conversion of DHA to MGO. Variations in antimicrobial activity for the lower dilutions of both vegan honea with DHA and DHA alone suggest that the sugar in the Honea, or other ingredients, might be influencing the activity observed or conversion of DHA to MGO. In the vegan Honea samples, there is higher sugar content causing a more acidic and dehydrating environment, potentially influencing the conversion reactions of DHA to MGO [27]. Importantly, the highest concentration of vegan honea with DHA and DHA alone both showed bactericidal activity, demonstrating MGO generation and the ability to generate antimicrobial activity.

On the other hand, the vegan Honea with DHA stored at 4 °C exhibited very little growth inhibition compared to storage at 37 °C and no bactericidal activity. This highlights that slower conversion of DHA to MGO occurs at lower temperatures. It also highlights that the indirect mechanisms of Honea might be impacting the antimicrobial activity since DHA alone was not inhibitory. Furthermore, the accelerated conversion of DHA to MGO at 37 °C meant that the DHA alone control exhibited antimicrobial activity, further indicating the higher temperature accelerates DHA to MGO conversion. However, at 4 °C the concentrations were not bactericidal, alluding to other influences on the antimicrobial activity than DHA to MGO conversion and indirect mechanisms alone. Furthermore, it has been suggested that other factors might influence the conversion of DHA to MGO that were not addressed here, such as proton donors/acceptors that influence the change of DHA from a dimer to a monomer, a necessary step for MGO conversion [27].

The combination of adding glucose oxidase and invertase to Honea containing DHA resulted in the same inhibition as observed for the addition of glucose oxidase and invertase alone. This was largely attributed to the high rate of hydrogen peroxide production from the addition of the enzymes, causing a high production of hydrogen peroxide resulting in any potential influence of MGO generation being masked. It is possible that with more controlled hydrogen peroxide generation, the combination of hydrogen peroxide and MGO generation together with the pressures of the high sugar content could result in more improved antimicrobial activity.

A limiting factor of this study was the absence of MGO detection throughout. The industry standard for MGO detection in manuka honey is through high performance liquid chromatography or mass spectrometry, which was unfortunately unachievable for this study. Therefore, the concentrations of MGO achieved in the vegan Honea could not be determined, so the full extent of DHA to MGO conversion and therefore the impact on the antimicrobial activity cannot be fully elucidated. However, it is well established that both DHA and MGO are present in manuka honey and that conversion from DHA to MGO occurs continuously over time. Therefore, a measurement would only be absolutely accurate for one time point, and is more an indicator of relative abundance of manuka components rather than a fixed absolute number. Controlling the amount of DHA is thus more informative in terms of the potential limits of MGO. Furthermore, the increase in antimicrobial activity over time shows the increasing presence of an antimicrobial component. This is significant because DHA alone on day 0 did not inhibit *M. abscessus* and further indicates MGO generation was occurring. Additionally, 5 mM MGO was required to inhibit *M. abscessus* (Fig 1B), suggesting a significant amount of MGO generation had to occur to cause antimicrobial activity. This provides a promising basis for further developing honey products and understanding important mechanisms behind their action. Furthermore, eukaryotic cell cytotoxicity studies were not conducted for either the vegan Honea with H_2_O_2_ generated or MGO generated, another consideration that is imperative for future applications.

Importantly, we have demonstrated that through the addition of the precursors that give rise to the antimicrobial activity of honey in a non-honeybee derived vegan Honea alternative, antimicrobial activity can be achieved. In the case of *M. abscessus,* we have shown that high sugar content alone is not enough to inhibit the growth but with the addition of these components bactericidal activity can be achieved. This is a significant finding, demonstrating that a synthetic honey is not only possible but could be a much improved alternative to regular honey. Furthermore, it demonstrates efficacy against *M. abscessus*, a highly drug resistant opportunistic pathogen. The possibility of engineering a targeted honey therapy against pathogenic bacteria is an exciting prospect, and should be explored further as a new focus of antimicrobial honey research.
